# Spatiotemporal Variation and Hotspot Detection of the Avian Influenza A(H7N9) Virus in China, 2013–2017

**DOI:** 10.3390/ijerph16040648

**Published:** 2019-02-22

**Authors:** Zeng Li, Jingying Fu, Gang Lin, Dong Jiang

**Affiliations:** 1 College of Geoscience and Surveying Engineering, China University of Mining & Technology, Beijing 100083, China; lizeng_cumtb@163.com; 2 State Key Laboratory of Resources and Environmental Information System, Institute of Geographical Sciences and Natural Resources Research, Chinese Academy of Sciences, Beijing 100101, China; fujy@igsnrr.ac.cn; 3 College of Resources and Environment, University of Chinese Academy of Sciences, Beijing 100049, China; 4 Key Laboratory of Carrying Capacity Assessment for Resource and Environment, Ministry of Land &Resources, Beijing 100101, China

**Keywords:** H7N9, spatial-temporal characteristics, hotspot analysis, SaTScan

## Abstract

This study aims to describe the spatial and temporal characteristics of human infections with H7N9 virus in China using data from 19 February 2013 to 30 September 2017 extracted from Centre for Health Protection of the Department of Health (CHP) and electronic databases managed by China’s Center for Disease Control (CDC) and provincial CDCs synthetically using the Geographic Information System (GIS) software ArcMap™ 10.2 and SaTScan. Based on the multiple analyses of the A(H7N9) epidemics, there was a strong seasonal pattern in A(H7N9) virus infection, with high activity in the first quarter of the year, especially in January, February, and April, and a gradual dying out in the third quarter. Spatial distribution analysis indicated that Eastern China contained the most severely affected areas, such as Zhejiang Province, and the distribution shifted from coastline areas to more inland areas over time. In addition, the cases exhibited local spatial aggregation, with high-risk areas most found in the southeast coastal regions of China. Shanghai, Jiangsu, Zhejiang, and Guangdong were the high-risk epidemic areas, which should arouse the attention of local governments. A strong cluster from 9 April 2017 to 24 June 2017 was also identified in Northern China, and there were many secondary clusters in Eastern and Southern China, especially in Zhejiang, Fujian, Jiangsu, and Guangdong Provinces. Our results suggested that the spatial-temporal clustering of H7N9 in China is fundamentally different, and is expected to contribute to accumulating knowledge on the changing temporal patterns and spatial dissemination during the fifth epidemic and provide data to enable adequate preparation against the next epidemic.

## 1. Introduction

The first human infection with avian influenza A(H7N9) was observed in Shanghai in March 2013 [1]. It posed a great pandemic threat to humans; previously the A(H7N9) virus had been detected only in birds. Due to its high pathogenicity and prevalence, the novel H7N9 virus was defined as “an unknown threat” by WHO (World Health Organization) and has attracted much attention [2]. During the past five years, the avian influenza virus has evolved and acquired mutations in the processes of continuous transmitted infections. Several experiments in 2016 revealed that some avian influenza virus strains have acquired the ability to adhere to recipient cells located in the human upper respiratory tract [3,4]. As a result, the strains have the potential to severely aggravate the transmission and spread of the virus among humans. Some researchers found that the virus’ protease cleavage site in the host has changed and that such variant viruses might be highly pathogenic in poultry. These variant strains have been identified in two patients with A(H7N9) virus infection in Guangdong Province [5]. In October 2017, the first discovery of the highly pathogenic H7N9 virus was reported by Chen after a long period of surveillance data collection and experimental studies [6]. He believed that the virus evolved rapidly, as in the case of the new strain of H7N9, based on its frequent gene recombination and susceptibility to mutation in the propagation process. If exposed to this virus, a human’s body would likely present a strong driving force to cause pathogenic mutations. In response the serious situation presented by the virus, a large number of experiments and much vaccine research on the novel H7N9 virus were conducted in 2015 and 2016. However, the ongoing increase in the number of A(H7N9)-virus-infected people in the current fifth A(H7N9) epidemic has aroused widespread public concerns [7,8]. 

There are many challenges to face in responding to this threat, including the frequent occurrence of cases, the high fatality rate, the unknown and potentially intractable genetic variation of the virus, the limitations to medical technology, the potential for pandemics, and the unpredictability of the virus. These issues have become increasingly prominent [9]. The A(H7N9) virus represents an unprecedented challenge to human survival. Understanding the basic laws of epidemics and the spatial-temporal cluster characteristics of avian influenza is crucial to improving public health security and informing governments for pandemic preparedness [10].

There is substantial research showing that the outbreak and spread of avian influenza follow basic rules: (1) In the early stage of transmission, the A(H7N9) virus is strongly concealed. Scientists are rarely recruited to detect typical symptoms in poultry until they are transported to other cities, even those carrying the virus [11]. (2) Due to exhibiting epidemic features (periodicity and seasonality) on the time scale, human cases of avian influenza A(H7N9) virus infection have emerged annually during the winter–spring period and peaked in the spring in Mainland China since 2013 [12]. (3) The spatial distribution of H7N9 cases shows that the areas from near the Yangtze River delta (YRD) to farther south around the Pearl River delta (PRD) have the highest densities of H7N9 cases and form strong space–time concentrated areas [10]. (4) The records of most patients indicate exposure to poultry before infection [13]. (5) Although some previous studies investigated the spatiotemporal clusters of H7N9 by SaTScan in China, the study periods were not long, with most studies concentrated on the period between 2013 and 2014 [14,15]. Considering the dynamic development process of influenza A(H7N9) human infections [16] and expanding beyond previous studies (i.e., collecting adequate cases to study the epidemic trends in different phases) [17], we collected data on the latest cases covering all four seasons of almost five years to investigate the periodical spatiotemporal distribution of H7N9.

The number of cases peaked in 2014 and generally decreased in subsequent epidemics up to a strong rebound in A(H7N9) cases in December 2016. Some researchers found that the fifth epidemic started earlier in the year spread to more regions and counties in affected provinces and had more confirmed cases than previous epidemics before 1 October 2016 [18]. The strong prevalence and spatial aggregation of cases in both time and space have drawn public concern over whether the virus poses an increasing pandemic threat since the fifth A(H7N9) epidemic [19]. The lack of an appropriate assessment of the prevalence of the avian influenza A(H7N9) virus and the unknown route of transmission may facilitate a potential pandemic and have devastating impacts on health and the economy worldwide. 

China is the original site of the virus outbreak and the most severely affected area in the world. Chinese people have suffered severely from the avian influenza A(H7N9) virus over the years. The Chinese government has been working to establish an effective system of disease surveillance and to monitor the epidemic. In addition, the statistical department of the Chinese government is collecting detailed and up-to-date data on infections around the world. This study provides the most comprehensive and up-to-date detailed data on human infections with A(H7N9) virus over five outbreaks in China. This study aims to conduct multiple analyses of the H7N9 epidemics to increase understanding of the current features of the epidemiological distribution of the virus and to explore its temporal and geographical patterns, especially its spatial-temporal clustering. The findings are expected to contribute to accumulating knowledge on the changing temporal patterns and spatial dissemination during the fifth epidemic and provide data to enable adequate preparation against the next epidemic.

## 2. Data and Methods 

### 2.1. Data Acquisition

We established a multi-source database on laboratory-confirmed human cases of A(H7N9) virus infections. It included basic information such as the event ID, patient age, patient sex, date, clinical symptom severity, affected area, latitude and longitude, and activation record of every case. We collected this information mainly from avian influenza weekly reports produced by the Centre for Health Protection of the Department of Health (CHP) and electronic databases managed by China’s Center for Disease Control (CDC) and provincial CDCs. Local CDSs are required to conduct a field investigation for each A(H7N9) virus infection and report aggregated data to a national surveillance system. China’s CDC is responsible for publishing the data in standard form. CHP is responsible for extracting data on both humans and birds and for highlighting global avian influenza activity. 

Uniform-format data contribute to analyses and exploration of characteristics. To eliminate the inconsistency between the onset date and the report date, we selected the time at which the patient was identified as infected with the A(H7N9) virus and was reported to the local CDS by the hospital as the date in this study. The diversity of multiple-source data increases the complexity of processing spatial attributes and increases the margin of error. Here, the administrative levels defining prefecture-level cities were used as the default values. Province-controlled divisions were regarded as administrative cities. The total number of patients in prefecture-level cities were compiled, including all counties and districts. Sometimes it was difficult for officials to identify the specific site of the infection, such as when the infected person frequented different cities before seeking medical advice or before infection was confirmed. Under such circumstances, after referring to the literature and consulting experts, the hospital location was temporarily assigned as the outbreak location [15]. We extracted the center coordinate point of the administrative city, and the WGS1984 coordinate system was adapted in this study. The study covered the period from 19 February 2013 to 30 September 2017, which included all of the epidemics in China.

### 2.2. Methodology

GIS analysis of the human infection database was conducted for the entire study area. The Spatial Autocorrelation and Getis-Ord Gi* (Getis-Ord Gi* statistic (pronounced G-i-star) calculated in Hot Spot Analysis) tool of the software ArcGIS10.2 (Environmental Systems Research Institute, Inc., Redlands, CA, USA) was used in the study. This tool provided intuitive visual presentations of results regarding the distribution of infections and spatial clusters at various stages of historical outbreaks in Mainland China. Using this tool, Global Moran’s I, Getis-Ord Gi* statistic, and Moran’s I index value were calculated, and z-scores and p-values were calculated to examine the null hypothesis that the attribute under analysis was randomly distributed among the regions [20]. These results were used to evaluate whether the A(H7N9) cases were clustered in a statistically significant manner across the whole study area. To test for statistically significant local clusters and to determine the general spatial extent of such clusters, we used the Getis-Ord Gi* statistical tool [21,22]. The Getis-Ord Gi* statistic is useful for differentiating clusters of influenza A(H7N9) human cases with high values from those with low values. Moreover, clusters of human cases that occur randomly can also have an influence on the spread of an infectious disease. 

Spatial-temporal analysis, as an epidemiological method, can extract and mine information from epidemic data. The SaTScan model adapts the spatial-temporal interactive scan and statistics method to detect the cluster characteristics of cases. Relevant date and location information is the only required inputs in the space-time permutation version of the model [23]. When or where the number of observed cases in a certain area is greater than the expected, a higher risk of the disease is indicated. To date, many scholars have carried out experiments to simulate the spreading process of diseases and have attempted to identify the aggregative time period and hotspot regions using the SaTScan model. 

In the model, a cylindrical window corresponding to space at the base and to time in the vertical direction is moved in space and time. The cylinder is centered at a county with various spatial radii to search for clusters and expands in height with different temporal values [24]. The cylinder modifies its shape to fit the increasing number of cases and the changing period of the unit center. The method is based on dynamic programming of the cylinder windows over scanning area and time. Finally, the method identifies significant clusters in both the spatial and temporal dimensions. In our study, space-time permutation was selected to run both in both purely spatial and purely temporal clusters. The number of replications was set to 999 times to search the high-rate areas. The maximum cluster size was set to 10% of the population at risk. The time aggregation length was set to 7 days, as was the maximum time aggregation. For the specific research methods, please refer to the supplementary material.

## 3. Results

### 3.1. Characterization in Time

The circulation period of the avian influenza A(H7N9) virus starts from 1 October of each year and ends on 30 September of the following year. Taking the novel strains of the A(H7N9) virus into consideration, the first epidemic period continues from February 2013 to 30 September 2014. The following four cycles follow the standard pattern. By the end of 30 September 2017, the database contained a total of 1558 cases of clinically confirmed infections reported by the government. The percentages of male and female patients in this infection group were 69.9% and 30.1%, respectively. Young people accounted for 67.7% of cases. The underage and older groups had lower incidences of infection with percentages of 3.6% and 5.5%. Elderly females represented only 24 cases. 

**Figure 1:**
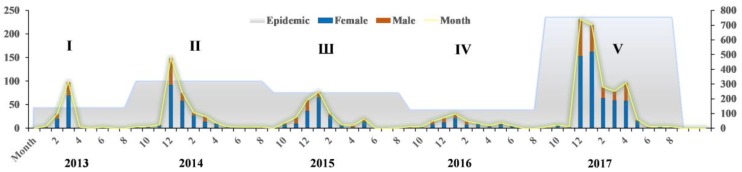
Weekly and monthly numbers of cases of human infection with avian influenza A(H7N9) virus by gender.

Figure 1 shows a fluctuating curve and bars of the number of cases by epidemic and monthly interval. At the scale of epidemic cycle, the bar chart displays two highs and two lows. There was a sudden outbreak in 2013, with a peak in number during the second epidemic. The number of cases then decreased after 2014, reached a historic low at the fourth epidemic, and then peaked again at the end of the studied period. It is remarkable that 316 cases arose during the second period, a number almost triple that in 2013. In the next two years, the number dropped steadily due to effective prevention and isolation measures of the Chinese government. The affected population gradually decreased to an all-time low (119 cases) in the fourth epidemic. The fifth epidemic, which ended on 30 September 2017, is represented by 751 laboratory-confirmed cases. These cases represent 48.2% of all infections, with this epidemic being the largest to date. At the monthly scale, A(H7N9) infections continue for 58 months, with an average of 26 cases per month. The black curve shows regularity in the timeline because of the virus’ seasonal appearance. The numbers typically increased to a peak in the first quarter of each year, especially in January and February. The numbers then declined significantly in the second quarter. The numbers then decreased to minimal levels or even to zero when entering the third quarter. Infections then reappeared in September and increased slowly during the fifth quarter. A rapid increase was observed around the transition from December to January of the next year. Fluctuations in number were observed among the periods. Overall, the highest incidence occurred in January and February 2017, with both months exceeding 200 cases. The number of A(H7N9) infections in January and February was 8.9 and 8.4 times the average monthly value, respectively. The next highest numbers of cases were observed for January 2014, with 149 cases, and April 2013, with 97 cases. There were no people affected in June, August, or September 2013, in July or August 2015, or in August or September in 2016 due to high temperatures. Compared with the corresponding month of the previous years, each month of 2017 had a significantly higher number of infections, with the peak value of the past five years occurring in this year. 

**Figure 2:**
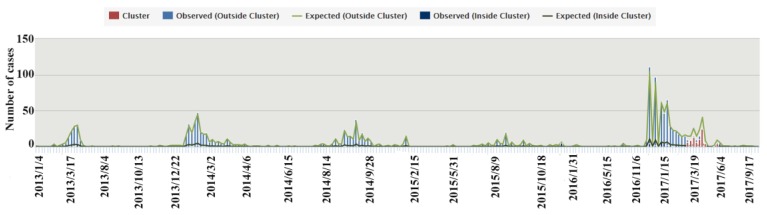
Weekly case numbers of human infection with the avian influenza A(H7N9) virus.

For the entire studied period, the weekly number of H7N9-infected individuals was also calculated, as shown in Figure 2. It was found that, for the primary cluster, the observed number within the cluster was greater than the expected number. In addition, the greatest number of infections broke out in the fifth period, from April to June. The periods of secondary clusters had similar observed-to-expected ratios outside the cluster, which were smaller than 1. These time periods showed high probabilities of aggregation. They were irregularly distributed from 2013 to 2017, particularly in the winter–spring transition.

### 3.2. Characterization in Space

**Figure 3:**
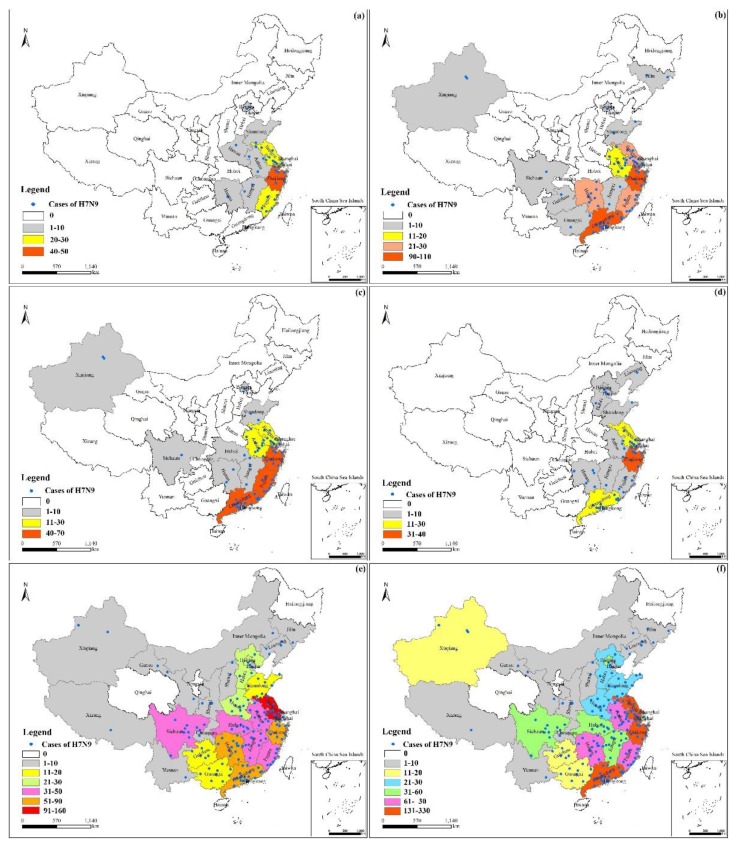
The distribution and the provincial statistics of human infection with avian influenza A(H7N9) virus in China. (**a**–**f**) represent the first phase epidemic, the second phase epidemic, the third phase epidemic, the fourth phase epidemic, the fifth phase epidemic, and all epidemics in the past five phases, respectively.

The avian influenza A(H7N9) virus caused various levels of damage in the 28 provinces and 183 prefecture-level cities in Mainland China, as shown in Figure 3. The results showed clear spatial differences among different areas. As shown in Figure 3f, cases of A(H7N9) infection were significantly more abundant in some regions than in others, with higher frequencies in the coastal provinces of Southern China than in the inland provinces. The highest concentrations of cases occurred in Eastern China, especially in the vicinity of the Yangtze River Delta, including Zhejiang Province, Jiangsu Province, and Fujian Province. The total number in these three provinces represented a 46% proportion of the cases in Mainland China.

As shown in Figure 3a–e, the virus’s ability to spread increased over time, with increased spreading into more inland areas. The continually increasing number of infections and the emergence of infections in new places were partially corroborated. The number of cases in Hunan Province grew consistently after 2013, increasing by an average of 144%. There were 7 inland areas in which the A(H7N9) virus newly emerged in 2017, including Inner Mongolia Autonomous Region, Gansu Province, Shaanxi Province, Shanxi Province, Chongqing Municipality, Tibet, and Yunnan Province, which are areas of Western and/or Northern China. However, Shanghai Municipality was an exception. It is the only city in which the number of infections dropped steadily over the years, including the most serious fifth epidemic. 

**Figure 4:**
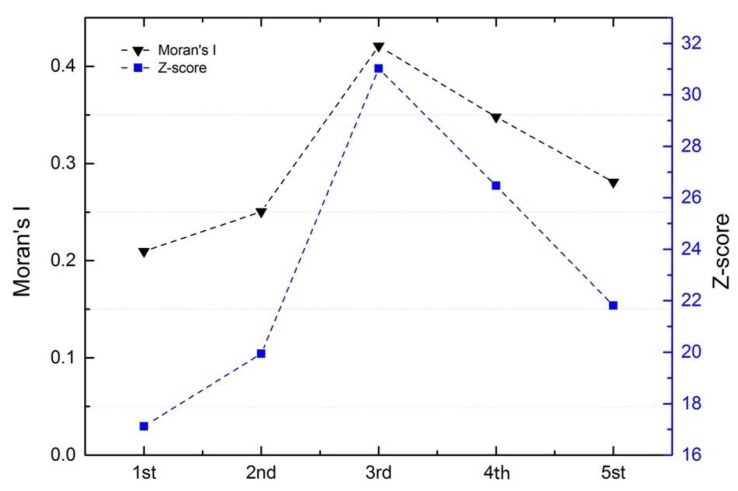
Moran’s I index and Z-score values in I-V epidemic areas.

Here, we conducted a GIS analysis using the A(H7N9) database and spatial autocorrelation. Moran’s I index was calculated to identify any clustering in the spatial locations of infections in the past five epidemics. As shown in the Figure 4, the value of Moran’s I index in each epidemic phase was greater than 0, indicating a global positive correlation (*p* < 0.0001). The value of the third epidemic phase had the maximum index value (0.4208), indicating the highest level of clustering. Although most cases occurred in the fifth epidemic phase, the distribution was scattered; thus, the Moran’s I index value was not the highest observed. Across all five epidemics, the Moran’s I index value was 0.0820, the Z-score was 5.6341, and the *p*-value was 0.0000, indicating significant clustering of the cases.

### 3.3. Characterization of Hotspots

**Figure 5:**
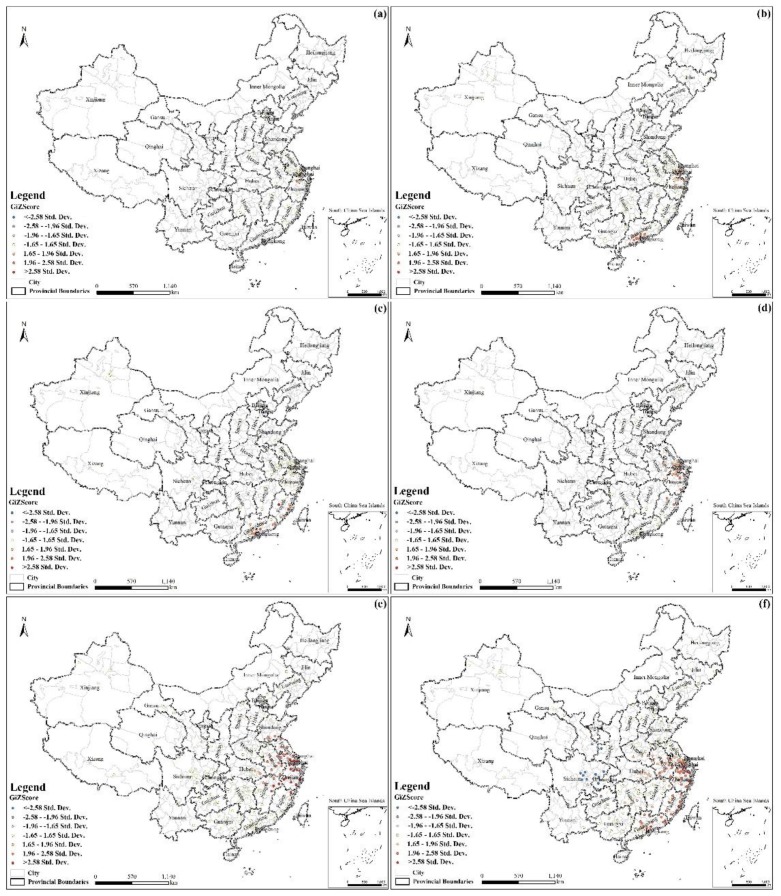
Maps showing the hotspot and cold spot for influenza A(H7N9) human cases in China. (**a**–**f**) represents the first phase epidemic, the second phase epidemic, the third phase epidemic, the fourth phase epidemic, the fifth phase epidemic, and all epidemics in the past five phases, respectively.

As shown in Figure 5, the distribution of hotspots changed over time. The scale in the figure indicates the Gi Z score: the red dots represent hotspots; the higher the value, the greater the spatial aggregation of high values (hotspots). The blue dots represent cold spots; the lower the value, the greater the spatial aggregation of low values (cold spots). Areas of random distribution are shown in yellow. As shown in the map in Figure 5a, there is a hotspot in the north of Zhejiang, and counts are few in the first epidemic. As shown in the map in Figure 5b, the number of hotspots had significantly increased and diffused gradually, with most located in Shanghai, the north of Zhejiang and the middle of Guangdong. A(H7N9) broke out during this period in Guangdong. As shown in the map in Figure 5c, there was no hotspot in Zhejiang, but infections had spread to Fujian, and the hotspots were mainly distributed in the coastal areas of Guangdong and Fujian. As shown in the map in Figure 5d, the hotspots were mostly located in Shanghai and Zhejiang, with a few spots in the east of Anhui and the north of Fujian. These data show that the epidemic was gradually spreading over time. The map in Figure 5e shows the most hotspots and the largest area of affected sites, mostly located in Shanghai, Zhejiang, Jiangsu, Anhui, the north of Jiangxi and Fujian, the east of Hubei, and the southwest of Shandong. In the map in Figure 5f, which covers all epidemics, the hotspots are mostly located in the southeast coastal areas of China, including the ten provinces of Shanghai, Jiangsu, Zhejiang, Guangdong, Fujian, Anhui, Henan, Hubei, Hunan, and Jiangxi. The distribution of hot spots expanded as the disease spread.

### 3.4. Characterization of Time-Space Clustering

**Figure 6:**
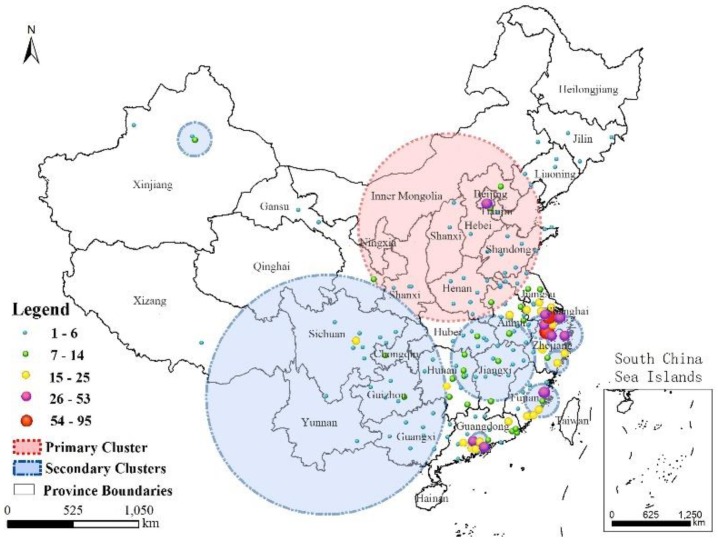
Distribution of the clusters and the occurrence frequency of the A(H7N9) virus in space.

**Figure 7:**
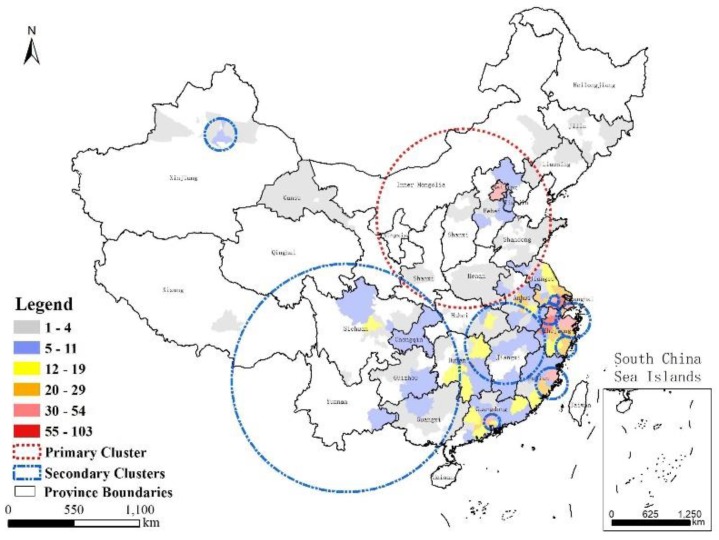
Areas of the clusters and cities affected by the A(H7N9) virus.

As shown in Figure 6, the SaTScan results identified 11 clusters with statistical significance (*p*-values less than 0.05) in Mainland China, with one primary cluster and 10 secondary clusters. The single primary cluster was located in Northern China and covered the Beijing-Tianjin-Hebei region, Shanxi Province, Shaanxi Province, Henan Province, and Shandong Province. The clustering time spanned from 9 April to 24 June 2017, and 79 cases were involved. The secondary clusters were used to detect the most likely aggregation patterns in local regions or time periods after the primary cluster scanning. It was therefore expected that they might overlap areas of other secondary clusters or the primary cluster. The secondary clusters had extremely wide geographical distributions and scattered time periods, accounting for 511 cases. The 10 secondary aggregation areas were mainly distributed in the southern coastal areas, especially in Eastern China and a small part of Southern China. Most of them were distributed in the 2013 and 2017 time frames. The model did not consider data from Jilin Province, Liaoning Province, Gansu Province, or Tibet, because these regions failed to meet the requirement of only sporadic infections in some restricted periods. Furthermore, Tibet and Gansu Provinces exhibited a long geographical span in an east–west direction. This pattern was different from that in Midwestern Jiangsu Province and Eastern Guangdong Province. There was a large group of A(H7N9) infections in each epidemic. The highly decentralized incidence in different cities was a concern in this analysis, although they accumulated in a continuous time span. Evaluation of the historical infected areas along with the spatial information of the clusters revealed that they represented 61.5% of the number of cities in the whole study area. As shown in Figure 7, the primary cluster encompassed 34 prefecture-level cities with infections. Due to aggregation in 2017, the first-level region possessed numerous newly affected areas with frequencies lower than 11. The other, secondary clusters involved 70 cities with various frequencies. Strong spatial clustering characteristics and an extended time period facilitated the formation of hotspot zones in some local areas; for example, Cluster ID 6 covered the major part of Hangzhou (a total of 103 cases). 

## 4. Discussion

Our study provides a comprehensive description of the epidemiology of laboratory-confirmed cases of A(H7N9) virus infection during the last five epidemics, presents the temporal and spatial dissemination pattern of A(H7N9) epidemics in Mainland China, and tests the cluster characteristics of patients infected with A(H7N9) in time and geography.

### 4.1. Analysis of The Epidemiological Characteristics of Human H7N9 Cases

A previous study indicated a higher risk in the cool months, especially when the minimum temperature is between 5 and 9 °C and the maximum temperature is between 13 and 18 °C [25]. Although there is not sufficient information to determine specific relationships or the impact of virus activity in the timeline, the analyses suggested that there may be some correlation between outbreaks and time. Some medical organizations and departments have conducted research to identify a solution and/or prevention measures. To date, an inactivated virus vaccine, a live attenuated vaccine, and a recombinant vaccine have been investigated in a few clinical studies involving Vero cell cultures and ASO3 and MF59 adjuvants, but much work remains before a vaccine can be entered into production [26,27].

Geographic data normally show autocorrelation, rather than being independent. It is the same with avian influenza A(H7N9) data. Spatial autocorrelation methods are effective ways to measure the spatial interdependence of geographic data [28]. However, correlation analysis is unable to produce precise results when incidents form aggregated areas and cannot provide information on the size or coverage of such areas. The Gi* statistic was adopted in this study to recognize hotspots of A(H7N9) human cases based on the case points. With this method, the infections exhibited local spatial aggregation, with hotspot areas found mostly in the southeast coastal area of China. This epidemiological trend may be related to population factors, such as local population density, as well as temperature, precipitation, relative humidity, the distribution of poultry, and other factors. As we do not explore these factors in this paper, further work should be conducted to study the influences of relevant factors. The space-time sequence probability model is one type of SaTScan model and is suitable for testing hypotheses [29]. We combined this model and other methods together in the current study to provide a comprehensive, multi-perspective, multi-dimensional geographic analysis of avian influenza A(H7N9) infections. The approaches detected the degree of spatial autocorrelation (Moran’s I) and defined high-frequency time periods and epidemic-prone geo-locations over all of the epidemics, which were beneficial for mining and analyzing the information in the geographic database of A(H7N9) infections.

### 4.2. Limitations

Some studies have shown that the spatial density of A(H7N9) virus infections in waterfowl and poultry is positively correlated with the incidence of A(H7N9) infections in humans [30,31,32,33]. Artois et al. recently concluded that poultry predictor variables became much more important in the last two epidemic waves than they were previously, supporting the assumption of much wider H7N9 transmission in the chicken reservoir [34]. There is a wide variety of poultry transactions in coastal areas. Our results based on the SaTScan model yield the consistent conclusion that a greater number of people living in Eastern China are infected with the A(H7N9) virus. Our study has several limitations. The database provides supporting data for medical research but does not provide evidence of the pathway of the virus and whether the epidemic can be spread among people. In addition, poultry consumption is only one of the indicators used to evaluate the A(H7N9) infections [35]. Factors such as poultry rearing and slaughter, the migration pathway of poultry products, and the uncertain transmission of the virus may be important drivers affecting future global prevalence. However, in this study, we did not account for the impacts of such factors [36,37]. Here, we collected data on cases of A(H7N9) infection in Mainland China and did not establish a database comprehensive in global scope. 

## 5. Conclusions

Our study constructed a comprehensive, up-to-date geographic database of the avian influenza A(H7N9) infections and conducted spatial-temporal analyses of five consecutive epidemics. The following primary conclusions were attained:

(1) The fifth epidemic started earlier in the year and affected more individuals and more districts than did the previous four epidemics based on analyses of the timeline and geographical distribution. Cases shifted from coastline areas to more inland areas over time.
(2) The cases exhibited local spatial aggregation, with high-risk areas most found in the southeast coastal regions of China. Shanghai, Jiangsu, Zhejiang, and Guangdong were the high-risk epidemic areas, which should arouse the attention of the local governments.
(3) Combining GIS and SaTScan methods increased our understanding of the development of A(H7N9) virus epidemics. A strong cluster from 9 April to 24 June 2017 was identified in Northern China, and there were many secondary clusters in Eastern and Southern China, especially in Zhejiang, Fujian, Jiangsu, and Guangdong Provinces.

The up-to-date database of avian influenza A(H7N9) cases and the temporal-spatial assessments of the epidemiological characteristics can help prevention in the next pandemic outbreak. However, the problem is complex, and further analysis, especially from the perspectives of space and time expansion, is warranted.

## Supplementary Materials

The following are available online at https://www.mdpi.com/1660-4601/16/4/648/s1.
