# Supplementary material for: Spatiotemporal Variation and Hotspot Detection of the Avian Influenza A(H7N9) Virus in China, 2013–2017
Source: Int J Environ Res Public Health. 2019 Feb 22;16(4):648. doi: 10.3390/ijerph16040648 (PMC6406651; doi:10.3390/ijerph16040648)
Supplement: Supplementary file 1 [file ijerph-16-00648-s001.pdf]

Methodology

1. Global Moran's I

Moran's I Index statistic was used for the measurement of spatial autocorrelation[1]. Significance of the index is assessed using both the z-score and P-value. The values of Moran's I range from −1 to +1, and Moran's $I > 0$, $= 0$, and < 0 indicate positive spatial autocorrelation, random distribution, and negative spatial autocorrelation, respectively[2]. The z-score was used to decide whether to reject the null hypothesis, and the probability of a false rejection was tested by the p-value[3]. Moran's I has been widely used in epidemiology, including in studies on haemorrhagic fever[5], human brucellosis[6], and the under-five mortality rate[7]. Moran's I adopts a covariance term between each point and its neighbours as follows:

$$I = \frac{N}{S_0} \times \frac{\sum_{i=1}^{n} \sum_{j=1, j \neq i}^{n} w_{ij}(x_i - \bar{x})(x_j - \bar{x})}{\sum_{i=1}^{n} (x_i - \bar{x})^2} \quad (1)$$

$$S_0 = \sum_{i=1}^{n} \sum_{j=1}^{n} w_{ij} \quad (2)$$

where n is the total number of cases; $W_{i,j}$ is the spatial weight between the cases i and j; x_i and x_j are the numbers of A(H7N9) cases in the ith and j^{th} points, respectively; and W_{ij} is the spatial neighbourhood weight for points i and j. The weight is defined based on adjacent neighbours as shown in the following equation [5],

$$w_{ij} = \begin{cases} 1 & \text{If } i, j \text{ are adjacent neighbours} \\ 0 & \text{otherwise} \end{cases} \quad (3)$$

afterwards, the weight matrix is standardized by row, i.e., every neighbour weight for a point is divided by the sum of all neighbour weights.

2. Hotspot Detection and Analysis

Global indices do not specify the location of cluster(s). To test for statistically significant local A(H7N9) clusters and to determine the general spatial extent of those clusters, we used the Getis-Ord Gi* statistical too[8]. The Getis-Ord Gi* statistic was used to identify A(H7N9) clusters of high values from clusters of low values. Moreover, clusters of cases that occur randomly can also have an influence on the spread of an infectious disease[2]. The Gi * statistic is written as follows[9]:

$$Gi^* = \frac{\sum_{j=1}^{n} w_{i,j} x_j - \bar{X} \sum_{j=1}^{n} w_{i,j}}{S \sqrt{\frac{n \sum_{j=1}^{n} w_{i,j}^2 - \left(\sum_{j=1}^{n} w_{i,j}\right)}{n-1}}} \quad (4)$$

$$\bar{X} = \frac{\sum_{j=1}^{n} x_j}{n} \quad (5)$$

$$S = \sqrt{\frac{\sum_{j=1}^{n} x_j^2}{n} - (\bar{X})^2} \quad (6)$$

where x_j is the number of A(H7N9) cases in the area j, $w_{i,j}$ is the spatial weight between points i and j, and n is the total number of points.

The Gi* statistic is a z-score, and therefore, no further calculations are required. The output from the Gi * statistic identifies spatial clusters of high values (hot spots) and spatial clusters of low values (cold spots) and provides confidence level bins (Gi_Bin) with features in the +/-3; +/-2; and +/-1 bins statistically significant at the 99%, 95%, and 90% confidence levels, respectively. Spatial aggregation for features with 0 for the Gi_Bin field was not statistically significant[10].

3. Spatiotemporal Permutation Scan Statistics

In this research, the spatiotemporal permutation scan statistic was used in the SaTScan software version 9.5, which is freely available from www.satscan.org[12]. The spatiotemporal permutation model introduced by Kulldorff was applied to analyse a space-time featured variable[13]. This model does not require population-at-risk data and can be used for the early detection of disease outbreaks when only the number of cases is available. Scan statistics are used in a retrospective way to detect past clusters using retrospective data and in a prospective way to detect clusters at the present time [11]. Scan statistics are explained by a cylindrical window with a circular geographical basis and the height indicating time. The window moves in space and time and therefore covers each potential time span for each geographical location resulting in defining an infinite number of overlapping cylinders of different forms and sizes that finally cover the entire study area.

The Poisson generalized likelihood ratio was used to estimate the likelihood of a cluster in a given spatiotemporal cylinder. Finally, Monte Carlo permutation was used to test for the significance level of clusters. In the model, a cylindrical window corresponding to space at the base and to time in the vertical direction is moved in space and time. The cylinder is centred at a county with various spatial radii to search for clusters and expands in height with different temporal values[12]. The cylinder modifies its shape to fit the increasing number of cases and the changing period of unit centre. The method is based on dynamic programming of the cylinder windows over scanning area and time. Finally, the method identifies significant clusters in both the spatial and temporal dimensions. In our study, space-time permutation was selected to run both in both purely spatial and purely temporal clusters. The number of replications was set to 9 999 times to search the high-rate areas. The maximum cluster size was set to 10% of the population at risk. The time aggregation length was set to 7 days, as was the maximum time aggregation.

References

1. Anselin L, Getis A. Spatial statistical analysis and geographic information systems. *Annals of Regional Science* 1992;26(1):19-33.

2. Bhunia GS, Kesari S, Chatterjee N, et al. Spatial and temporal variation and hotspot detection of kala-azar disease in Vaishali district (Bihar), India. *Bmc Infectious Diseases* 2013;13(1):64.

3. Ahmadkhani M, Alesheikh AA, Khakifirouz S, et al. Space-time epidemiology of Crimean-Congo hemorrhagic fever (CCHF) in Iran. *Ticks Tick Borne Dis* 2017;9(2)

4. Abbas T, Younus M, Muhammad SA. Spatial cluster analysis of human cases of Crimean Congo hemorrhagic fever reported in Pakistan. *Infect Dis Poverty* 2015;4(1):9.

5. Mollalo A, Alimohammadi A, Khoshabi M. Spatial and spatio-temporal analysis of human brucellosis in Iran. *Trans R Soc Trop Med Hyg* 2014;108(11):721-28.

6. Li Z, Fu J, Jiang D, et al. Spatiotemporal Distribution of U5MR and Their Relationship with Geographic and Socioeconomic Factors in China. *International Journal of Environmental Research & Public Health* 2017;14(11):1428.

7. Ord JK, Getis A. Local Spatial Autocorrelation Statistics: Distributional Issues and an Application. *Geographical Analysis* 1995;27(4):286-306.

8. Ma LG, Chen QH, Wang YY, et al. Spatial pattern and variations in the prevalence of congenital heart disease in children aged 4-18 years in the Qinghai-Tibetan Plateau. *Science of the Total Environment* 2018;627:158-65.

9. ESRI. Hot Spot Analysis (Getis-Ord Gi*)—Help | ArcGIS for Desktop [Internet] ArcGis for desktop2018 [updated Consulted May 2018. Available from: http://resources.arcgis.com/zh-cn/help/main/10.2/index.html#/na/005p00000011000000/ accessed Consulted May 2018.

10. Blanco-Guillot F, Castañeda-Cediel ML, Cruz-Hervert P, et al. Genotyping and spatial analysis of pulmonary tuberculosis and diabetes cases in the state of Veracruz, Mexico. *PloS one* 2018;13(3):e0193911.

11. Kulldorff M, Heffernan R, Hartman J, et al. A space–time permutation scan statistic for disease outbreak detection. *PLoS medicine* 2005;2(3):e59.

12. Kulldorff M. SaTScan-Software for the spatial, temporal, and space-time scan statistics. *Boston: Harvard Medical School and Harvard Pilgrim Health Care* 2010.
